# Preliminary study on the assessment of visceral adipose tissue using dual-energy x-ray absorptiometry in chronic obstructive pulmonary disease

**DOI:** 10.1186/s40248-016-0070-3

**Published:** 2016-10-04

**Authors:** Francesca De Blasio, Erica P. A. Rutten, Emiel F. M. Wouters, Luca Scalfi, Francesco De Blasio, Marco A. Akkermans, Martijn A. Spruit, Frits M. E. Franssen

**Affiliations:** 1 Department of Research and Education, CIRO, Horn, The Netherlands; 2 Department of Public Health, “Federico II” University of Naples Medical School, Naples, Italy; 3 Respiratory Medicine and Pulmonary Rehabilitation Section, Clinic Center, Private Hospital, Naples, Italy

**Keywords:** Body composition, COPD, DEXA, Fat mass, Visceral adipose tissue

## Abstract

**Background:**

Visceral adipose tissue (VAT) was shown to be increased in patients with chronic obstructive pulmonary disease (COPD) compared to control subjects with comparable body mass index (BMI). Our aim was to determine the relation of VAT by dual-energy x-ray absorptiometry (DEXA) in patients with COPD by disease severity, BMI, other indices of body composition and static lung volumes.

**Methods:**

294 COPD patients admitted for rehabilitation were studied. Lung function, static lung volumes and body composition (i.e. BMI, waist circumference, fat-free mass, fat mass and fat distribution between android and gynoid fat mass) were assessed before entering pulmonary rehabilitation. VAT was estimated within the android region by using DEXA. Patients were stratified for gender, BMI (cut-off of 25 kg/m^2^) and GOLD stage. To assess the impact of VAT on lung volumes, patients were also stratified for VAT less and above 50^th^ percentile.

**Results:**

Both male and female patients with more severe airflow limitation had significantly lower VAT values, but these differences disappeared after stratification for BMI. VAT was significantly and strongly correlated with other body composition parameters (all *p < 0.001*). Patients with moderate to severe airflow limitation and lower VAT had increased static lung hyperinflation and lower diffusing capacity for carbon monoxide. Nevertheless, multivariate stepwise regression models including for BMI, age, gender and forced expiratory volume in 1 s (FEV_1_) as confounders did not confirm an independent role for VAT on static lung hyperinflation and diffusion capacity.

**Conclusion:**

After stratification for BMI, VAT is comparable in moderate to very severe COPD patients. Furthermore, BMI and demographics, but not VAT, were independent predictors of static lung hyperinflation and diffusing capacity in COPD.

## Background

Although defined by the presence of persistent airflow limitation [1], alterations in body weight and body composition contribute to disease severity in patients with chronic obstructive pulmonary disease (COPD). Indeed, low body mass index (BMI) (<21 kg/m^2^) is related to poor exercise tolerance [2], low fat-free mass (FFM) [3] and co-morbid osteoporosis [4]. Furthermore, it is incorporated in the prognostic assessment of COPD [5]. Otherwise, high BMI (≥30 kg/m^2^) is related to increased dyspnea [6], reduced weight-bearing exercise performance [7] and systemic inflammation in COPD. [3] At the same time, reductions in lung volumes have been consistently reported in obese COPD patients [8].

In addition to the discriminating role of BMI in the clinical phenotyping of COPD patients, alterations in body composition, i.e. the distribution between FFM and fat mass (FM), are common and have additional impact on the burden of disease. In more detail, visceral adipose tissue (VAT) is receiving increasing attention in COPD, since it was shown that elderly subjects with obstructive lung disease have increased visceral adipose area in comparison with control subjects with comparable BMI [9]. Also, there is increasing evidence for a role of VAT in the pathophysiology of systemic inflammation [10] and cardiovascular morbidity in COPD [10, 11]. Typically, VAT is assessed by computer tomography (CT) or magnetic resonance imaging (MRI), but these measurements are expensive, time-consuming and not widely available. Alternatively, dual-energy x-ray absorptiometry (DEXA) is a commonly used device with lower radiation dose, alternative to estimate body composition in COPD and was recently introduced as a validated method to quantify VAT [12, 13]. However, no clinical studies investigating VAT with DEXA are available in COPD patients. Thus, associations between VAT assessed by this method and other traditional markers of body composition in COPD are currently unknown. Also, the impact of VAT *per se* on lung volumes has not been studied in COPD.

Therefore, the aims of the present study were: 1) to quantify the amount of VAT measured by DEXA in patients with COPD in relation to disease severity and BMI; 2) to explore the relationships between VAT and other indices of body composition and 3) to study the impact of VAT on static lung volumes. In the present study, we hypothesized that VAT does significantly and negatively influence static lung volumes and hyperinflation in patients with COPD.

## Methods

### Patient selection

Data were abstracted from the Integrated Knowledge System based on BioXM™ (Biomax Informatics AG, Munich, Germany) of 294 patients with a primary diagnosis of COPD [post-bronchodilator forced expiratory volume in 1 s (FEV_1_) /forced vital capacity (FVC) ratio of ⩽0.70 and post-bronchodilator FEV_1_ < 80 % predicted] who were evaluated during the initial assessment of a comprehensive pulmonary rehabilitation (PR) program at CIRO Horn (The Netherlands) between August 2014 and May 2015 [14]. Exclusion criteria were: a history of asthma, α1-antitrypsin deficiency, lung cancer, any previous lung surgery, instable inflammatory or endocrine diseases, acute myocardial infarction within the last 6 months, any known bone disease other than osteoporosis, treated malignant disease within the last 5 years and an exacerbation of COPD within the last 4 weeks. Ethical approval was not indicated because all of the tests were done as part of the routine initial assessment [14], and analyzed retrospectively. The Board of Directors of CIRO approved the use of de-identified patients’ records.

### Lung function measurement

As part of the routine 3-day initial assessment of integrated health status, all patients performed post-bronchodilator spirometry (Masterlab®; Jaeger, Würzburg, Germany). FEV_1_ and FVC were assessed in accordance with the latest GOLD guidelines [1]. Inspiratory vital capacity (IVC) was assessed too. Plethysmographic lung volumes, such as Total Lung Capacity (TLC), Intra Thoracic Gas Volume (ITGV), Expiratory Reserve Volume (ERV) and Residual Volume (RV) were assessed. Inspiratory Capacity(IC)/TLC ratio [15] was calculated by dividing TLC − ITGV for TLC, and was used as a marker for hyperinflation (defined as an abnormal increase in the volume of air remaining in the lungs at the end of spontaneous expiration [16]). Diffusing capacity of the lung for carbon monoxide (DL_CO_) was measured using the single-breath method. All obtained values are expressed as percentages of the predicted value, by comparison with age and sex-specific reference values [17].

### Body composition

**Fig. 1:**
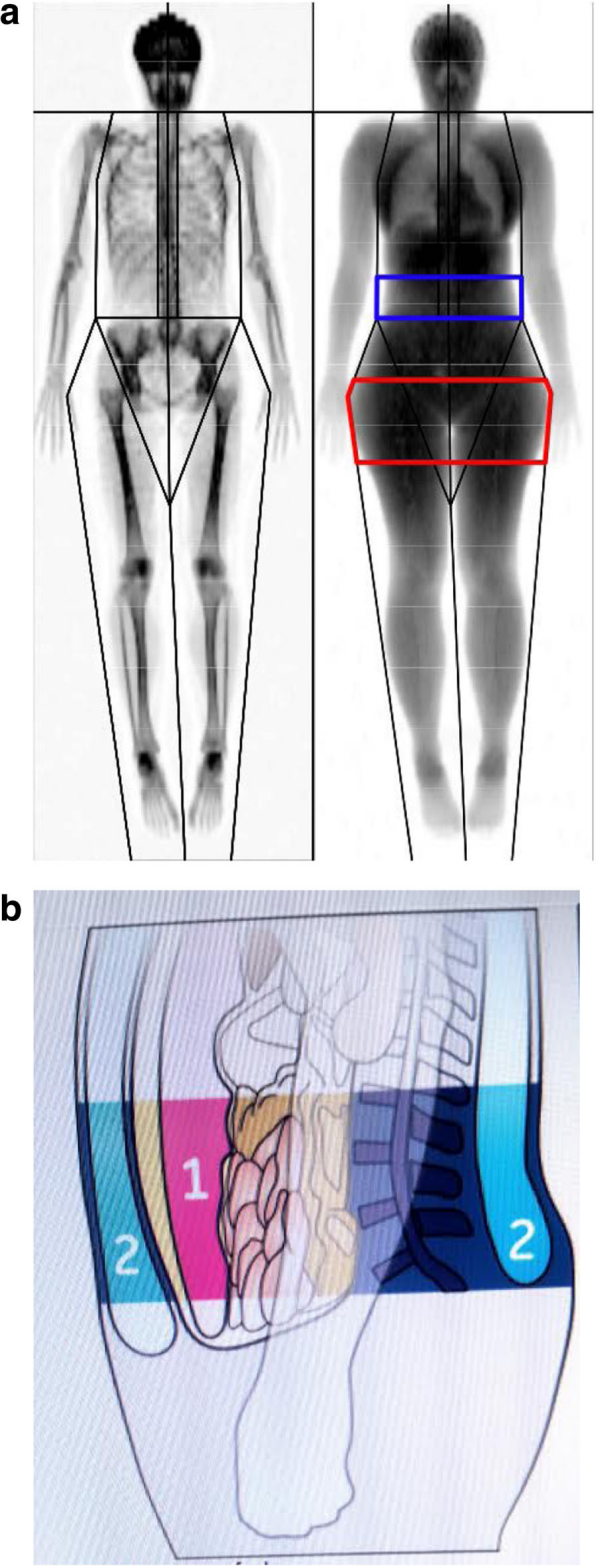
**a** A model of total body DEXA scan. The android region is highlighted in blue and the gynoid region is highlighted in red (refer to text for further explanations). **b** Representation of visceral adipose tissue (1) and subcutaneous adipose tissue (2) within the android region

Body height was measured to the nearest 0.5 cm with a wall-mounted stadiometer. Body weight was assessed to the nearest 0.1 kg using a weighing scale, and BMI was calculated as weight/height^2^. A total body scan was performed by DEXA using a Lunar Prodigy® system (GE Healthcare - enCORE v14, Madison, WI, USA). Subjects lay supine on the DEXA table with arms adequately separated from the trunk and were instructed to remain still throughout the scanning procedure. After analysis of the whole body scan, a quadrilateral box was manually drawn around the L1–L4 region of interest (abdomen) bounded inferiorly by the horizontal line identifying L4/L5 vertebral space and superiorly by the horizontal line identifying the T12/L1 vertebral space. Scans were displayed with an adjustment of the gray scale, so that all of the soft tissue in the designated area was included. From the total body scan, body composition was assessed: bone mineral content (BMC), lean mass (LM), FFM (LM plus BMC). FFMI was calculated by dividing FFM by height^2^, and FM by subtracting FFM from total weight. The base of the android region (Fig. 1a - shown in blue) sits immediately above the pelvis and is in height equal to 20 % of the distance from the pelvis to the chin. The android and gynoid (Fig. 1a - shown in red) regions are separated by a distance equal to 1.5 times the height of the android region, while the height of the gynoid region is double that of the android one. VAT was estimated within the android region [12] (Fig. 1b number 1).

#### Statistical Analysis

Values are reported as means ± SD with the exception of continuous variables, presented as median and interquartile range (IQR). In order to quantify the amount of VAT in relation to disease severity, patients were stratified for gender, BMI (cut-off of 25 kg/m^2^) and GOLD stages (I + II, III, IV). Since the proportion of patients with mild airflow limitation referred for PR is limited [18], these patients were grouped with those with moderate airflow limitation. Furthermore, in order to study the impact of VAT on lung volumes, both men and women were divided in two subgroups according to VAT 50^th^ percentile (2183.0 g for men and 912.5 g for women).

Statistical analysis was performed using SPSS version 20.0. Comparisons across GOLD stages were performed using ANOVA with post-hoc Tukey-Kramer tests for multiple comparisons. Subsequently, multivariate stepwise linear regression analysis was performed to investigate a relationship between VAT and selected indices of lung hyperinflation. A *p* <0.05 was considered significant in all analyses.

## Results

### Subjects characteristics

Patients were characterized by moderate to very severe airflow obstruction (GOLD I/II, 44.9 %; GOLD III, 38.1 %; GOLD IV, 17.0 %). 154 (52.4 %) were men (median age 67 years, IQR 13 years; median BMI 26.9 kg/m^2^, IQR 8.6 kg/m^2^) and 140 (47.6 %) were women (median age 61 years, IQR 13 years; median BMI 25.3 kg/m^2^, IQR 8,1 kg/m^2^). The median number of pack/ years smoked was 40 (IQR 24), 30.3 % of patients were current smokers and 63.6 % of patients were former smokers. 25.5 % of patients used long-term oxygen therapy.

### VAT across GOLD stages

**Fig. 2:**
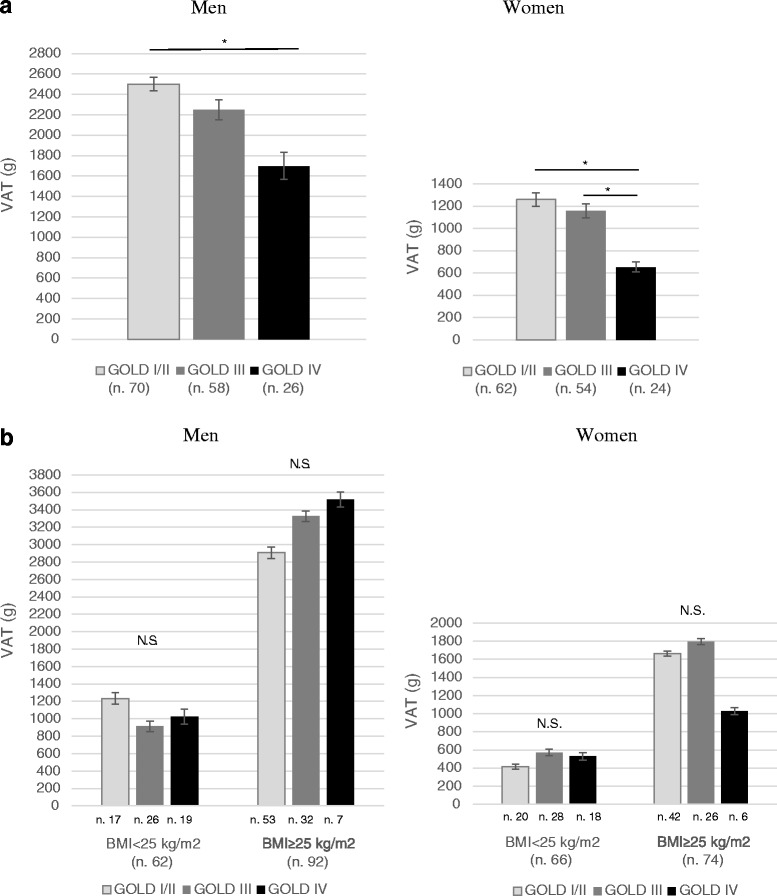
**a** Visceral adipose tissue (g) in COPD male (n. 154) and female (n. 140) patients across GOLD stages, before stratification for BMI. Mean values and standard error are reported. *: p  < 0.005. **b** Visceral adipose tissue (g) in COPD male (n. 154) and female (n. 140) patients across GOLD stages, after stratification for BMI. Mean values and standard error are reported. *: p  < 0.005

The amount of VAT separated for men and women is shown in Fig. 2a. In each GOLD subgroup VAT was greater in men than in women (in all GOLD stages: *p < 0.001)*. Furthermore, patients with more severe airflow obstruction had significantly lower values of VAT, both in men (GOLD I/II: 2501.1 g ± 1141.7 g; GOLD III: 2246.4 g ± 1488.5 g; GOLD IV: 1697.2 g ± 1350.1 g, *p* =0.031) and women (GOLD I/II: 1259.7 g ± 938.0 g; GOLD III: 1160.0 ± 929.8 g, GOLD IV: 653.0 ± 442.8 g, *p* =0.015). Figure 2B shows the amount of VAT in the three different GOLD subgroups after stratification for BMI: as it could be expected, patients with a BMI ≥ 25 kg/m^2^ had consistently higher values of VAT, compared with those with a BMI < 25 kg/m^2^ (*p* < 0.001), both in men and women. After stratification for gender and BMI, differences in VAT across the three GOLD subgroups disappeared (Fig. 2b).

### Relationships of VAT with other indices of total and central adiposity

**Fig. 3:**
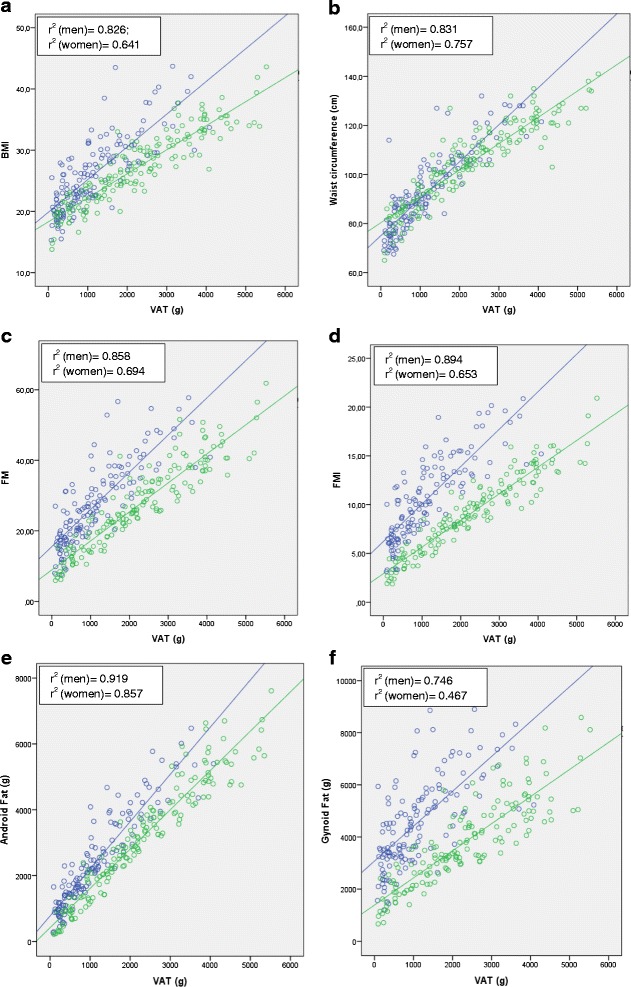
Correlation plots of VAT with BMI (**a**), waist circumference (**b**), FM (**c**), FMI (**d**), android fat mass (**e**) and gynoid fat mass (**f**). *P* were <0.001 for each correlation, in both genders (o (Blue) = men; o (Green) = women)

The amount of VAT was significantly and strongly correlated with BMI, waist circumference, FM, FMI, android fat mass and gynoid fat mass (Fig. 3 a-f). The strength of correlations was higher in male than in female patients. Android fat mass was the most strongly correlated with VAT (r^2^ = 0.919 in men; r^2^ = 0.857 in women), followed by waist circumference (r^2^ = 0.831 in men; r^2^ = 0.757 in women), FM (r^2^ = 0.858 in men; r^2^ = 0.694 in women), FMI (r^2^ = 0.894 in men; r^2^ = 0.653 in women), BMI (r^2^ = 0.826 in men; r^2^ = 0.641 in women) and gynoid fat (r^2^ = 0.746 in men; r^2^ = 0.467 in women).

### Impact of VAT on lung volumes

Besides disease severity, patients were stratified for VAT less and above the 50^th^ percentile. Patients with moderate to severe airflow limitation and lower VAT levels had increased ITGV and ERV and reduced IC/TLC (Table 1) compared to those with higher VAT levels. There was no significant difference for FEV_1_ (% pred), FEV_1_/FVC (%) or IVC (% pred), or for lung volumes in GOLD IV patients.

**Table 1:** General and clinical parameters in 294 COPD patients stratified for GOLD stages and divided in two groups, based on VAT 50^th^ percentile (2183 g for men; 912.5 g for women)

	GOLD I + II (n. 132)			GOLD III (n. 112)			GOLD IV (n. 50)		
	VAT < 50^th^	VAT > 50^th^	*P*	VAT < 50^th^	VAT > 50^th^	*P*	VAT < 50^th^	VAT > 50^th^	*P*
Age (years)	65.3 ± 10.9	64.9 ± 9.2	*0.856*	65.1 ± 10.4	65.7 ± 6.8	*0.697*	59.6 ± 7.5	59,1 ± 10.5	*0.868*
BMI (kg/m^2^)	24.0 ± 3.5	31.9 ± 4.4	***<0.001***	21.6 ± 2.9	30.5 ± 4.8	***<0.001***	20.7 ± 3.4	28.1 ± 4.5	***<0.001***
FFMI (kg/m^2^)	16.7 ± 2.4	18.7 ± 2.6	***<0.001***	15.5 ± 1.7	17.9 ± 2.5	***<0.001***	14.6 ± 1.4	16.9 ± 2	***<0.001***
FMI (kg/m^2^)	7.5 ± 2.0	13.2 ± 3.0	***<0.001***	6.4 ± 2.4	12.7 ± 3.1	***<0.001***	6.2 ± 2.6	11.3 ± 2.9	***<0.001***
Gynoid Fat (g)	3322.2 ± 1005.5	5517.6 ± 1314.4	***<0.001***	2770.4 ± 1031.5	5148.4 ± 1572.9	***<0.001***	2810.2 ± 1251.9	4737.4 ± 1397.2	***<0.001***
Android Fat (g)	1797.3 ± 769.0	4120.8 ± 1110.6	***<0.001***	1428,9 ± 736.2	3997.5 ± 1274.8	***<0.001***	1336.6 ± 760.1	3641.8 ± 1513.6	***<0.001***
FEV_1_ (% pred)	70.6 ± 20.6	68.9 ± 13.5	*0.572*	38.5 ± 5.5	39.6 ± 5.7	*0.306*	23.4 ± 5.0	25.2 ± 3.1	*0.181*
FEV_1_/FVC (%)	46.8 ± 11.3	49.4 ± 9.6	*0.160*	31.5 ± 6.7	33.4 ± 7.4	*0.157*	22.9 ± 4.4	26.2 ± 7.3	*0.050*
IVC (% pred)	115.9 ± 19.5	108.1 ± 18.7	***0.021***	96.3 ± 17.0	93.0 ± 15.9	*0.289*	79.8 ± 15.4	78.4 ± 15.0	*0.750*
ITGV (% pred)	132.9 ± 27.6	112.5 ± 21.9	***<0.001***	163.6 ± 33.1	146.0 ± 27.1	***0.003***	193.4 ± 35.1	174.6 ± 37.6	*0.090*
ERV (% pred)	132.6 ± 46.4	106.9 ± 44.9	***0.002***	130.6 ± 40.1	112.2 ± 35.1	***0.012***	106.4 ± 29.8	90.4 ± 35.9	*0.103*
RV (% pred)	134.0 ± 33.7	116.1 ± 26.9	***0.001***	178.3 ± 40.3	159.3 ± 32.6	***0.008***	234.5 ± 61.8	212.9 ± 48.8	*0.226*
IC/TLC (%)	37.9 ± 8.2	42.6 ± 7.2	***0.001***	26.6 ± 7.7	30.2 ± 6.5	***0.008***	20.3 ± 5.4	24.0 ± 5.2	***0.027***
DLCO (% predicted)	54.8 ± 15.9	65.5 ± 22.6	***0.002***	42.7 ± 12.9	50.6 ± 12.5	***0.003***	33.6 ± 13.1	45.1 ± 16.4	***0.015***

Values reported as mean ± standard deviation

*BMI* body mass index, *FFM* fat-free mass index, *FMI* fat mass index, *FEV*_1_ forced expiratory volume in 1 s, *FVC* forced vital capacity, *IVC max* inspiratory vital capacity, *ITGV* intra-thoracic gas volume, *ERV* expiratory reserve volume, *RV* residual volume, *IC* inspiratory capacity, *TLC* total lung capacity, *DLCO* diffusing capacity of the lung for carbon monoxide

Statistically significant *p* are indicated in bold characters

The same trend was observed in both genders, although it was less pronounced in female than in male patients (data not shown). DL_CO_ was significantly increased in patients with greater VAT in all GOLD categories (*p* = 0.002 in GOLD I + II; *p* = 0.003 in GOLD III; *p* = 0.015 in GOLD IV). The same finding was true when considering male and female patients separately.

### Determinants of hyperinflation

In order to investigate whether VAT itself is a predictor of lung hyperinflation, independently from BMI, age, gender and FEV_1_ (L), stepwise regression was performed and results are shown in Table 2. Considering IC/TLC (%) as dependent variable (and FEV_1_ as the best predictor), the r^2^ of the regression model was 0.701. Only FEV_1_ (L), BMI, age and gender are included in the final equation and the multiple regression was significant (*p* < 0.001). VAT was excluded from the model.

**Table 2:** Multivariate stepwise regression with IC/TLC (%) as dependent variable

Model	Standardized Coefficients	Sig.
	Beta	
(Constant)		,000
FEV_1_ (L)	,710	,000
BMI	,289	,000
Age	-,091	,007
Gender*	-,092	,010

Independent variables: VAT, BMI, age, gender and FEV_1_

Adjusted R^2^ = 0.697. VAT was excluded from the model

*gender = 0 for women; =1 for men

Similar results were obtained when choosing ITGV (L) as dependent variable (Table 3). Only BMI, gender, FEV_1_ (L) and age were significant determinants, the r^2^ was 0.465 and the regression was significant (*p* < 0.001). VAT was again excluded from the model.

**Table 3:** Multivariate stepwise regression with ITGV (L) as dependent variable

Model	Standardized Coefficients	Sig.
	Beta	
(Constant)		,000
BMI	-,385	,000
Gender*	,505	,000
FEV_1_ (L)	-,391	,000
Age	-,133	,003

Independent variables: VAT, BMI, age, gender and FEV_1_

Adjusted R^2^ = 0.458. VAT was excluded from the model

*gender = 0 for women; =1 for men

Concerning diffusing capacity, stepwise regression results showed that FEV_1_ (L), age, gender and BMI were significant determinants of DL_CO_ (L) in the way that younger male patients and patients with higher BMI and higher FEV_1_ (L), have also higher DL_CO_ values (Table 4). Nevertheless, VAT was again excluded from the model. The r^2^ of the regression model was high (0.532), and the regression was significant (*p* < 0.001).

**Table 4:** Multivariate stepwise regression with DL_CO_ (L) as dependent variable

Model	Standardized Coefficients	Sig.
	Beta	
(Constant)		<0.001
FEV_1_ (L)	0.428	
Age	-0.232	<0.001
Gender*	0.239	<0.001
BMI	0.302	<0.001

Independent variables: VAT, BMI, age, gender and FEV_1_

Adjusted R^2^ = 0.525. VAT was excluded from the model

*gender = 0 for women; =1 for men

## Discussion

The present study reported several novelties regarding the research concerning the involvement of the adipose tissue in the pathology of COPD. First, it showed that VAT is comparable across GOLD stages for airflow limitation if BMI is taken into account. Second, traditional markers of total and central adiposity correlated very well with VAT assessed by DEXA. Third, VAT is not an independent predictor of static lung hyperinflation in COPD, while demographics and BMI are.

### VAT in COPD

Furutate et al. [19] investigated VAT in patients with COPD using CT. They observed that VAT was higher in COPD compared to controls with comparable BMI, and that VAT tended to increase with COPD progression, although not all differences were statistically significant. In addition, the authors found that, in patients with severe emphysema, VAT was retained despite the absence of obesity. In the same study, BMI, waist circumference, FFMI, FMI and SAT were shown to decrease with increasing emphysematous severity. Besides the fact that we did not relate VAT to emphysema severity, the differences in ethnicity (Furutate included a Japanese sample, the present study population were all caucasians) between studies makes comparison difficult, but our results indicates that VAT is comparable across GOLD stages, after stratification for BMI.

### Assessment of VAT in COPD

Several methods can be used for measuring VAT. The most commonly used imaging technique is abdominal CT, one of the current gold standards for quantifying VAT [20–24]. However, because of the radiation dose involved, the high costs and the heavily utilized clinical equipment requirement, CT is not optimal as screening method for VAT. MRI has also been used for VAT measurement [25, 26]. It avoids the radiation dose involved with CT measurements but still requires an expensive and time-consuming procedure as well as heavily utilized clinical equipment. DEXA is another suitable clinical reference method for the assessment of body composition. It measures the differential attenuation of two different energy level x-rays as they pass through the body and subdivides soft tissue into bone, lean and fat compartments. New software was recently proposed to quantify VAT in the android region by DEXA [12, 13], with several advantage relative to CT and MRI, such as lower costs, lower x-ray exposure and short-scanning time [27–30].

### Correlates of VAT

To date, no data are available on relationships between VAT measured by DEXA and other indices of body composition in COPD patients. In the present study we find a strong and significant correlation of VAT with BMI, waist circumference, FM, FMI as well as android and gynoid fat mass, in both genders but more strongly in males (likely due to their higher amount of VAT compared with females). As reported by Kaul et al., DEXA is a valid tool for quantification of VAT and other adipose tissue regions in the clinical setting [12], but more studies are needed in COPD in order to validate this method against CT, which is one of the current gold standards for quantifying VAT.

### Hyperinflation in COPD

To the best of the authors’ knowledge, this study is the first that carefully evaluated VAT by DEXA in relation to static lung volumes in a large group of patients with moderate to very severe COPD. O’Donnell et al. [31] examined the relationship between increasing BMI and plethysmographic lung volumes in a population with airflow obstruction. It was shown that lung hyperinflation decreased exponentially with increasing BMI. However, the potential impact of fat distribution on lung volumes was not investigated. In the present study, patients with greater VAT seemed to have lower ITGV, ERV, RV and higher IC/TLC and DL_CO_ without a difference in FEV_1_, FEV_1_/FVC or IVC in most GOLD stages (and most pronounced in early GOLD stage). This finding appears to confirm that the major effect of obesity is on lung volumes, with no direct effect on airway obstruction [32]. The results of the multiple stepwise regression analysis confirmed a predicted role of FEV_1_, BMI, gender and age on lung hyperinflation but no independent role of VAT for any of the lung volume parameters. This finding is somewhat surprising, since VAT is located in the abdominal region below the respiratory compartment. Future studies have to seek out what parameters of obesity are related to lung hyperinflation. Probably SAT in the abdominal and thoracic region could be worth considering.

In health, increasing BMI is known to be associated with increasing diffusing capacity, possibly reflecting the increased pulmonary blood volume in obesity [33, 34]. In the current study, a relationship was confirmed between increasing BMI and DL_CO_ in COPD patients. Nevertheless, no significant predictive role of VAT on DL_CO_ was found.

#### Limitations and strengths of the study

Various limitations of the study have to be considered. Firstly, there was no age-matched healthy control group included in the present study. Previous studies, however, already suggested that COPD patients have increased VAT by CT scan compared to matched controls. Secondly, no gold standard reference method (such as CT) was available and future studies need to validate the DEXA device for VAT. As mentioned in the introduction however, this was not the aim of the present study. Beyond these limitations, the major strength of this study was that it is the first study, which used DEXA in order to measure VAT in COPD patients. Additionally, a large population with moderate to very severe disease and careful clinical characterization was enrolled in the study.

## Conclusions

In conclusion, after stratification for BMI, VAT is comparable across GOLD stages in moderate to very severe COPD patients. VAT measured by DEXA is strongly correlated to other traditional and most commonly used body composition parameters, such as BMI, waist circumference as well as android and gynoid fat mass. BMI and demographics, but not VAT, were independent predictors of static lung hyperinflation and diffusing capacity in COPD.

### Clinical implications

VAT is now recognized as a risk factor for metabolic syndrome, diabetes, and cardiovascular disease [35–41]. Furthermore, since cardiovascular disease is one of the main comorbidities in COPD, together with type II diabetes, skeletal muscle dysfunction and osteoporosis [3], VAT is receiving increasing attention in clinical assessment of COPD patients.

DEXA provides lots of useful information in clinical practice. Not only is it the gold standard method for evaluating bone mineral density and discriminate patients with and without osteoporosis, but it is also a useful method for the assessment of other clinically relevant body composition parameters, among which VAT.

## Abbreviation

BMI, body mass index; COPD, chronic obstructive pulmonary disease; CT, computed tomography; DEXA, dual-energy x-ray absorptiometry; DLCO, diffusing capacity of the lung for carbon monoxide; ERV, expiratory reserve volume; FEV_1_, forced expiratory volume in 1 s; FFM, fat-free mass; FFMI, fat-free mass index; FM, fat mass; FVC, forced vital capacity; GOLD, global initiative for chronic obstructive lung disease; IC, inspiratory capacity; IQR, interquartile range; ITGV, intra thoracic gas volume; IVC, inspiratory vital capacity; MRI, magnetic resonance imaging; PR, pulmonary rehabilitation; RV, residual volume; SAT, subcutaneous adipose tissue; TLC, total lung capacity; VAT, visceral adipose tissue.
